# Does Polyvinylpyrrolidone Improve the Chemical Stability of Cilazapril in Solid State?

**DOI:** 10.22037/ijpr.2019.1100640

**Published:** 2019

**Authors:** Katarzyna Regulska, Miosz Regulski, Anna Wzgarda, Aleksandra Kotowska, Aleksandra Ignasiak, Barbara Ćwiertnia, Beata Stanisz

**Affiliations:** a *Greater Poland Oncology Center, 15* ^*th*^ * Garbary Street, 61-866 Poznań, Poland. *; b *Chair and Department of Toxicology, Faculty of Pharmacy, Poznan University of Medical Sciences, 30* ^*th*^ * Dojazd Street, 60-631 Poznan, Poland.*; c *Chair and Department of Pharmaceutical Chemistry, Faculty of Pharmacy, Poznan University of Medical Sciences, 6* ^*th*^ * Grunwaldzka Street, 60-780 Poznan, Poland.*; d *Chair and Department of Inorganic and Analytical Chemistry, Faculty of Pharmacy, Poznan University of Medical Sciences, 6* ^*th*^ * Grunwaldzka Street, 60-780 Poznan, Poland.*

**Keywords:** Cilazapril, Polyvinylpyrrolidone, Solid dispersion, Stability, Solid state

## Abstract

In this study a solid dispersion and a physical mixture of cilazapril (CIL) with a biopolymer - polyvinylpyrrolidone (PVP) as a carrier were prepared so as to investigate the effect of PVP on the stability of CIL. CIL is unstable in solid state and decomposes rapidly under humid conditions. It requires stabilization to ensure safety of its use. The studied CIL/PVP formulations were prepared by milling and evaporation technique. Their identity was confirmed by FT-IR method. The stability of CIL in the CIL/PVP formulations was assessed by forced ageing test under isothermic conditions using RP-HPLC. The influence of temperature (experimental conditions: RH 76.4% and T = 70, 75, 80, 85, and 90 ^o^C) and the effect of relative humidity (experimental conditions: RH 25.0%, 50.9%, 60.9%, 66.5%, 76.4%, T = 90 °C) on the rate of CIL degradation were examined. It was established that the process of CIL decay in the studied forms followed first-order kinetics with the formation of one degradation product - cilazaprilat. The degradation rate constant of this reaction was lower than that for pure CIL. The energy of activation of the CIL degradation in the presence of PVP was higher than that of pure CIL. Furthermore, CIL incorporated into PVP exhibited lower sensitivity to moisture. Based on these data PVP was considered as a potential stabilizing substance for CIL-containing dosage forms.

## Introduction

Quality assurance is one of the primary responsibilities of current pharmacy since it guarantees efficiency, safety, and economy of patient treatment. A fundamental aspect of pharmaceutical quality is drug stability which can be defined as the capability of a drug product to maintain its physio-chemical, microbiological, toxicological, and pharmacological properties above the established limits, throughout the pre-defined period, at specific storage conditions. Drugs, however, are continuously subjected to gradual degradation resulting in the final loss of their original features. Clinically, the described process impairs their quality as well as efficiency and safety of their use. Thus, there is a legal demand to determine the stability of pharmaceuticals not only during their research and development process but also in the post-approval management (1). 

The rate of drug degradation is considered as its individual attribute determined by the following factors: drug physio-chemical profile, the method of manufacture, and the storage conditions. Unfortunately, numerous pharmaceutical compounds fail to meet the criteria of stability and therefore they may not be introduced into clinical practice. Such agents need stabilization for example by appropriate packaging, co-formulation with stabilizers or by physio-chemical modification. Out these, the physical methods of maximizing drug stability seem particularly promising. They involve preparation of liposomes, formation of cyclodextrin complexes, and generation of physical mixtures and solid dispersions with biopolymers. Interestingly, in many studies the polymeric solid dispersions have demonstrated a significant advantage in the stabilization of amorphous and moisture-labile drugs providing a considerable improvement of their physical stability, solubility, and dissolution rate (2, 3). The following mechanisms behind the polymer-related drug stabilization have been identified so far: antiplasticization, specific intermolecular interactions, alteration of chemical potential, and reduction in molecular mobility. The technological methods of developing such systems involve: milling, evaporation, fusion, hot melt extrusion, freeze drying, spray drying, and supercritical fluid precipitation (2). 

One polymer commonly used in the technology of solid dispersions is polyvinylpirolidone (PVP). It occurs in the form of fine, white to creamy-white colored, odorless and very hygroscopic powder that absorbs water up to 40% of its weight from the atmosphere (4). A great advantage of this excipient is its low toxicity and the affinity to both: hydrophilic and hydrophobic molecules (5-7). It can also act as a drug stabilizer confirmed by Heidemann *et al.* in the preformulation studies, in which the enhanced stability of a drug in a formulation with hygroscopic excipient was shown. It was suggested that PVP preferentially binds water molecules leading to their reduced interaction with active ingredient (8). Furthermore, PVP was reported to form hydrogen bonds with moisture-sensitive drugs increasing thereby their solubility, dissolution rate, and stability (9). Such studies are available for the following drugs: celecoxib, chlorpheniramine, indomethacin, sulfonamides, naproxen, hydrocortisone, felodipine, nifedypine, reserpine as well as several model drugs (5, 9 and 10-19). In pharmaceutical industry PVP K-30 grade has been widely used (20). However, due to its high glass transition temperature the use of this polymer in the melt method is impossible, but its good solubility in most organic solvents makes it good for preparing solid dispersions by the solvent evaporation or milling (21).

Since PVP acts as efficient stabilizer of numerous moisture-labile drugs we decided to co-formulate it with cilazapril (CIL) which exhibits poor stability in solid state. CIL is a member of dicarboxylate-containing angiotensin-converting enzyme inhibitors (ACE-Is) - an appreciated group of pharmaceuticals used as first-line therapy in a wide array of cardiovascular-system related diseases, including: hypertension, symptomatic heart failure, diabetic and non-diabetic nephropathy as well as in the secondary prevention after acute myocardial infarction (22, 23). Our previous studies clearly indicated that CIL in the pure form (24, 25) as well as in the commercial pharmaceutical formulation (tablets) (26) is highly unstable and very sensitive to humidity and high temperatures. We have also found that several excipients, such as: hypromellose, lactose and talc significantly impair the stability of CIL while maize starch acts as its stabilizer probably due to the moisture-scavenging properties (26). Therefore, the stabilization of CIL by a non-costly and simple method seems reasonable and anticipated. In this study we decided to prepare a solid dispersion and a physical mixture of CIL and PVP by evaporation and milling technique.

## Experimental

*Materials*

CIL was kindly supplied by Biofarm, Poland (serial number 1621816). Methanol and acetonitrile were of HPLC grade (Merck, Darmstadt, Germany). Potassium phosphate monobasic and bromide, polyvinylpyrrolidone K30 grade (PVP) were purchased from Merck, Darmstadt, (Germany), ethanol 95% from Polmos S. A. (Poland). Water was freshly bidistilled.

All other chemicals were of analytical reagent grade.

*Preparation of CIL/PVP solid dispersion*

PVP (800 mg) and CIL (200 mg) were co-dissolved in 20.0 mL of methanol. Methanol was evaporated at 30 °C. The dry residue was crushed and grinded for 15 min into a fine powder in a mortar. 

*Preparation of CIL/PVP physical mixture*

CIL (200 mg) and PVP (800 mg) were crushed and grinded manually for 30 min in a mortar in order to obtain a fine powder.

*Identity confirmation by FT-IR*

Fourier Transform Infrared Spectrometer IRAffinity-1 (Shimadzu, Japan) was employed. Four KBr disco were prepared by compressing the mixture of 1.00 mg of each analyzed sample (*i.e.*: pure CIL, pure PVP and CIL/PVP physical mixture and CIL/PVP solid dispersion) with 300 mg of KBr in a Pye Unicam minipress. The spectra were recorded using KBr as blank. The measurement parameters were the following: range 400-4000 cm^-1^, resolution 4.0 cm^-1^, and the number of scans 40.

*Stability analysis; RP-HPLC apparatus*

High Performance Liquid Chromatograph Shimadzu, Japan was used. A chromatographic system consisted of a Shimadzu LC-6A Liquid Chromatograph pump with a 7725 Rheodyne value injector 20 µL fixed loop equipped with a Shimadzu SPD-6AV UV-VIS Spectrophotometric Detector. The detector was set at 212 nm and peak areas were integrated by Shimadzu C-R6A Chromatopac integrator. Columns (LiChroCART^®^ 250-4 mm HPLC-Cradridge, LiChrospher^®^ 100 RP-18 (5 µm) Merck, Darmstadt, Germany) worked at ambient temperature. They were eluted at the flow rate 1.5 mL min^-1^. The mobile phase consisted of acetonitrile, methanol, and phosphate buffer (pH 2.0) (60:10:30, v/v/v). Oxymetazoline hydrochloride was used as internal standard (IS) (24). 

Aqueous phosphate buffer at pH 2.0 was freshly-prepared by dissolving 0.0681 g of potassium dihydrogen phosphate (KH_2_PO_4_; M = 136.09 g mol^-1^) in 450 mL of bidistilled water. It was adjusted to pH 2.0 using ortho-phosphoric (V) acid (85%) and completed to 500.0 mL with bidistilled water.

*RP-HPLC – validation*

*Solution*

Stock solution was prepared by dissolving 20.00 mg of CIL in 50.0 mL of methanol. The solution was freshly prepared on the day of analysis and stored at 2 °C protected from light until used. After 7 days of storage it exhibited no evidence of decomposition. Standard solutions (ranging from 0.4 mg mL^-1^ to 0.08 mg mL^-1^) were prepared by diluting the stock solution with methanol. 

IS was prepared by dissolving 20.00 mg of oxymetazoline hydrochloride in 100.0 mL of methanol (c = 0.2 mg mL^-1^). After 7 days of storage at 2 °C protected from light it exhibited no evidence of decomposition.

*Calibration procedure*

The calibration curve for the HPLC analysis was determined by linear regression for both: CIL/PVP physical mixture and CIL/PVP solid dispersion. Ten standard solutions of pure CIL were prepared at the following concentrations: of 0.08; 0.12; 0.16; 0.20; 0.24; 0.28; 0.32; 0.36; 0.4 mg mL^-1^ (C_i_). Aliquots of 1.0 µL of each standard solution were taken, mixed with 0.5 µL of methanolic solution of IS and immediately injected into the chromatographic column. RP-HPLC analysis was conducted in triplicate with 25 µL-injections of each standard solution under the conditions described above. The relative peak areas P_CIL_/P_IS_ (P_CIL_ and P_IS_ – area of CIL and IS signals) were plotted versus the corresponding concentrations and the calibration curve was obtained. The regression equation was computed using the method of least squares.

*Precision*

Method’s precision is expressed as the relative standard deviation (RSD) of replicate measurements, while its accuracy is expressed by percentage of model mixtures recovery. In order to evaluate the repeatability (intra-day), eight replicate measurements for three different CIL concentrations (low c = 0.08 mg mL^-1^; medium c = 0.2 mg mL^-1^; high c = 0.4 mg mL^-1^) were performed on the same day using the proposed RP-HPLC method. The intermediate precision (inter-day) was studied by comparing the results obtained on two different days for CIL at the concentration of 0.2 mg mL^-1^.

*The limits of detection and quantification*

Limit of detection (LOD) and limit of quantification (LOQ) were calculated using the following formulae: LOD = 3.3S_y_/a and LOQ = 10S_y_/a, where S_y_ stands for the standard deviation of the blank signal and a is a slope of the calibration curve.

*Kinetic studies*

Forced ageing test was performed. The accurately weighed samples (0.0500 g) of CIL/PVP physical mixture and CIL/PVP solid dispersion were put into open, amber glass vials, and stored according to the following protocols:

*Estimation of temperature effect*

The prepared series of samples were incubated at 70 °C, 75 °C, 80 °C, 85 °C , and 90 °C under RH ~76.4% (obtained by the use of NaCl-saturated aqueous solution bath) in heat chambers with the temperature control accuracy of ±1.0 ^o^C.

*Estimation of RH effect*

The influence of RH was investigated under isothermal conditions within RH range of 25.0–76.4%. The following saturated salt baths were used to obtain the desired RH level: sodium iodide (RH ~25.0%), sodium bromide (RH ~50.9%), potassium iodide (RH ~60.9%), sodium nitrate (RH ~66.5%), and sodium chloride (RH ~76.4%). The appropriate solutions of inorganic salts were closed in desiccators and they remained in contact with the excess of solid salt throughout the study. The samples of CIL/PVP physical mixture and CIL/PVP solid dispersion were introduced into appropriate salt bath and inserted into the automatically controlled heat chamber set at 90 °C. In order to equilibrate the conditions of the kinetic test, the prepared salt baths had been incubated at the desired temperature for 24 h prior to the experiment.

*CIL/PVP concentration changes*

Within definite time intervals, determined by the rate of CIL degradation, the vials were withdrawn from the heating chambers, cooled to ambient temperature, dissolved in methanol, quantitatively transferred into volumetric flasks, made up with methanol to a total volume of 25.0 mL, and filtered (solution A). One milliliter of IS was added to 1.0 mL of each solution A (solution A_i_). The aliquots of 25 μL of the solutions A_i_ were injected onto the chromatographic column and the chromatograms were recorded. Basing on the remaining drug concentration (c) calculated from the measured relative peak areas (P_i_/P_I.S_.), the kinetic curves were constructed by the use of the method of least squares. In parallel, a comparative methanolic solution of pure CIL at the concentration of 0.8 mg mL^-1^ was prepared (solution B). One milliliter of the solution B was mixed with 0.5 mL of IS (solution B_i_). The aliquots of 25 mL of the solutions B_i_ were injected onto the chromatographic column. The obtained data were interpreted using the following relationship: P = P_CIL_/P_IS_ = f(t); where P_CIL_ is the area of CIL in CIL/PVP physical mixture and CIL/PVP solid dispersion while P_IS_ represents the signal for IS. The initial concentration of pure CIL in the samples (t = 0) was defined as 100%. The concentrations measured on the subsequent days were expressed as percentage of the initial concentration. The percentage of the content loss of CIL in CIL/PVP physical mixture CIL/PVP solid dispersion was calculated using the following equation: c (%) = (PA ∙ cB ∙ V)/(PB ∙ m), where: P_A_ = (P_CIL_/P_IS_); P_CIL_ – magnitude of CIL peak in the solution A_i_ of CIL/PVP physical mixture and CIL/PVP solid dispersion; P_B_ = (P_B1_/P_IS_); P_B1_ – magnitude of the pure CIL peak in the solution B_1_; P_IS_ – magnitude of IS; c_B_ – percentage concentration of the comparative pure CIL (c = 0.08%); V – volume coefficient (25); m – weighed amount of synthetic model mixture (m = 50.00 mg). The obtained results were then compared with those obtained for pure CIL in our earlier experiments (24, 25). 

*Identification of a degradation product*

The identification of a degradation product of CIL was carried out using a Waters liquid chromatographic system, interfaced to a 996 detector and a Micromass ZQ 2000 electrospray mass spectrometer. LC separations were made on column LiChrospher 100 RP-18 (size 10 mLm, 250 mm 4 mm) at temperature 30 °C. The flow rate of the mobile phase, which consisted of methanol – water – formaldehyde (49:50:0.5 v/v/v), was 0.5 mL min^-1^. The mobile phase was filtered through a filter (0.22 mm) and degassed by ultrasound. The injection volume was 100 mL. Recorded mass range m/z was from 100 to 1000, ionization ES^-^ was applied.

## Results and Discussion

The employed CIL/PVP ratio in solid dispersion (1:4) was selected based on the literature data and our previous experience (20, 21 and 27-29). The excess of PVP is necessary to obtain solid dispersion/physical mixture as well as the dissolution of the drug increases with the increased carrier content (27). However, the concentration of the drug must be high-enough to allow its identification. The obtained mixtures had satisfactory physical properties and their IR spectra and HPLC chromatograms exhibited well-separated lines with appropriate intensity. 

*The identity of CIL/PVP physical mixture and CIL/PVP solid dispersion*

**Figure 1:**
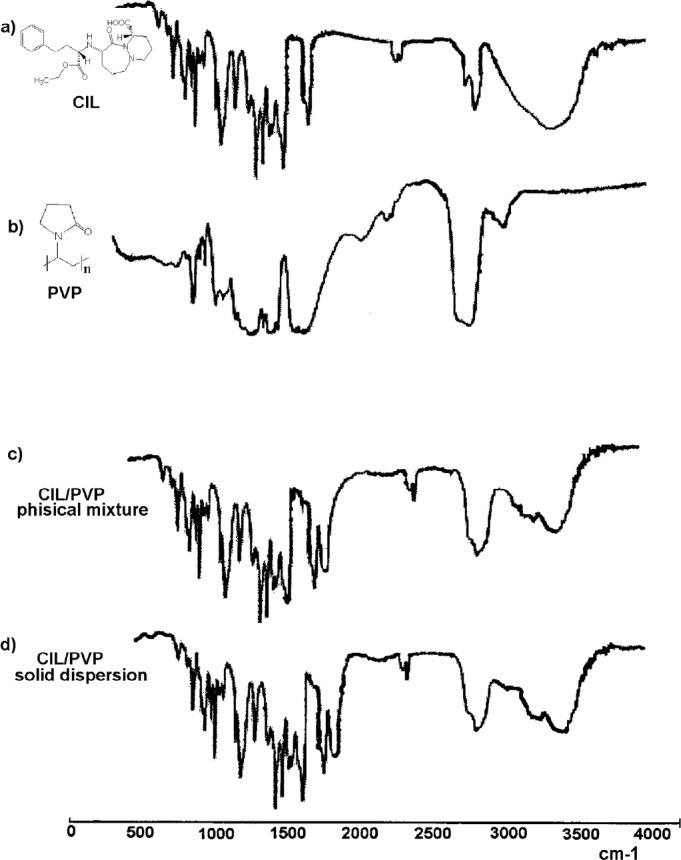
FT-IR spectra: (a) pure CIL, (b) pure PVP, (c) CIL/PVP physical mixture, (d) CIL/PVP solid dispersion

The identity of the prepared PVP/CIL solid dispersion and physical mixture was confirmed by FT-IR which was simple, rapid, and well-established (24). The recorded IR spectra are demonstrated in Figure 1. The scanned region of wavelengths contained absorption bands that characterize the entire molecular structure by vibrations of the spectrum. The spectrum for pure CIL (Figure 1A) exhibited the signals, which were attributed to the specific CIL functionals, *i.e.*: carboxyl -COOH – absorption band of stretching -C=O vibrations ѴC= O ~1900 cm^-1^, stretching -C-O vibrations ѴC -O ~1200 cm^-1^; aromatic and heterocyclic ring -stretching -C-N vibrations ѴC-N ~1100 cm^-1 , ^stretching -C=C vibrations ѴC=C ~1650–1450 cm^-1^, alkane and aromatic -C-H stretch vibrations ѴC - H ~3100–3000 cm^-1^, deformation vibrations δC-H~1200 – 950 cm^-1^ ; aliphatic functionals - ѴN-H – 3300 cm^-1^. The spectrum of pure PVP (Figure 1B) contained the following bands: stretch vibrations of carbonyl -C=O - ѴC=O ~1800-1900 cm-1 (a wide band), stretch vibrations of -C-O_ѴC-O _~1200- 1300 cm-1; for heterocyclic structure ѴC-N ~1100 cm-1 and for CH2 –2900-3000 cm-1 (a wide multiplet). The FT-IR spectra of CIL/PVP physical mixture and CIL/PVP solid dispersion (Figures 1C and 1D, respectively) turned out to look like the superimposed spectra of pure CIL and PVP. They display the bands attributed to pure CIL (ѴC=O _(ester and carboxyl)_ , ѴC-O _(ester and carboxyl)_, ѴO-H (wide multiplet), ΔO-H, ΔO-H; aromatic and heterocyclic ring - ѴC-N, ѴC=C, ѴC- H, ΔCH; aliphatic functional ѴC-H) and pure PVP (and pure PVP _C=O_ (a wide band), and pure PVP C-O; heterocyclic ring and pure PVP C-N and -CH2- functional (a wide multiplet). Furthermore, the similarity between the two spectra is visible. No additional bands of any new chemical groups were observed indicating that no chemical reaction in the obtained CIL/PVP systems occurred. This confirmed the applicability of PVP to serve as a carrier for CIL-containing physical mixtures and solid dispersions.

*Validation of RP-HPLC for kinetic study*

**Figure 2:**
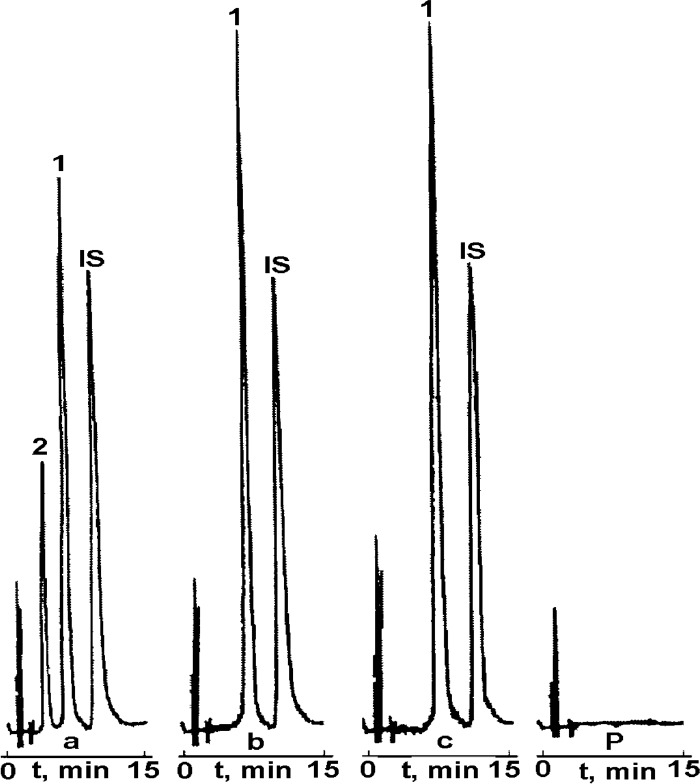
RP-HPLC chromatogram for: (a) CIL/PVP physical mixture (b) CIL/PVP solid dispersion, (c) pure CIL and (P) PVP. Retention times: CIL (1): tR ~7 min; degradation product (2): tR ~4 min; (IS) tR ~12 min

Prior to the main kinetic studies the validation of RP-HPLC method was performed in order to confirm that the employed procedure is suitable for the rapid, qualitative, and quantitative analysis of CIL/PVP degradation samples. This method had been successfully used before for the determination of pure CIL (24-26) as well as other structurally-related ACE-Is (30-39). In this study the following validation parameters were estimated: selectivity, linearity, precision and sensitivity, expressed as LOD and LOQ. The validation report was summarized in Tables 1 and 2. In the collected chromatograms for pure CIL and CIL/PVP samples (Figure 2) sharply-developed, well-separated peaks at reasonable retention times were observed indicating method’s good selectivity. They were attributed to CIL (t_R_ ~7 min), IS (t_R_ ~12 min) and the degradation product of CIL (t_R_ ~4 min). There were no signals of PVP in the time range of 2-15 min (Figure 2P) suggesting no interferences of the carrier in the performed HPLC process. The method was linear within the tested concentration range (0.08–0.4 mg mL^-1^) as indicated by the high correlation coefficient of the calibration curve (r = 0.998). The precision and accuracy were satisfactory (mean recovery around 100%) and the sensitivity (LOD = 0.025; LOQ = 0.073 mg mL^-1^; Table 1 was acceptable. Based on the positive validation results the method was accepted to further kinetic tests.

*The stability of CIL in CIL/PVP formulations*

The performed kinetic studies were based on the analysis of the RP-HPLC chromatograms generated for: CIL/PVP physical mixture (Figure 2A) CIL/PVP solid dispersion (Figure 2B), pure CIL (Figure 2C) and PVP (Figure 2P). 

**Figure 3:**
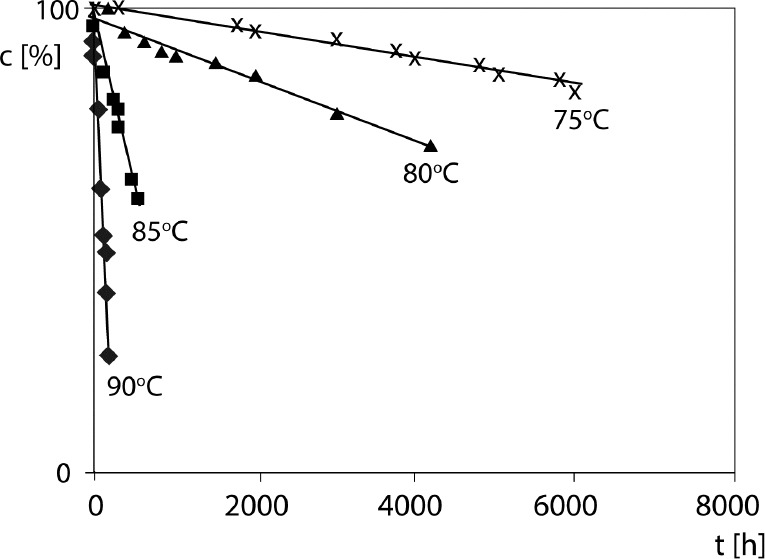
Semilogarithmic plot ct = f(t) for the degradation of CIL in CIL/PVP physical mixture (RH 76.4%, T 75 – 90 °C); First-order kinetics

The kinetic parameters (degradation rate constants k) and thermodynamic parameters (enthalpy of activation ΔH^≠^, entropy of activation ΔS^≠^ and energy of activation E_a_) of CIL decay were calculated.The results are depicted in Table 3. The kinetic order of CIL degradation in the formulation with PVP was established based on the amount of the remaining drug in the samples after their exposure to increasing temperature at elevated RH for different time intervals. This procedure is approved by ICH Guidelines for Industry Q1A (R2) stability testing of new drug substances and products. During the exposure the gradual loss of CIL content with the concomitant increase of the CIL degradation product was observed, while the appropriate kinetic curve c_t_ = f(t) took the form of exponential function. Hence, the studied reaction was defined as first order kinetics with one product. The rate of such reaction depends only on the concentration of CIL and it decreases linearly with the decrease of the reactant’s concentration. Taking the above into consideration the degradation rate constant of the observed reaction was calculated using the following formula: 

ln c_t_ = ln c_0_ – k^.^t

where: c_t_ (%) is the concentration of CIL in the sample during t [h] in the isothermal test, c_0_ is 100% and k [s^-1^] is the reaction rate constant. According to the theory of first-order kinetics the semilogarithmic plot c = f(t) is linear and its slope corresponds to the magnitude of the degradation rate constant k (Figure 3). Thus, the least squares method was used to calculate the regression parameters: y = ax + b, a ± ∆ a, b ±∆ b, standard errors S_a_, S_b_ and the correlation coefficient r. The ±∆ a, ±∆ b were estimated for f = n - 2 degrees of freedom and a=0.05 Table 3 summarizes the results for each experiment.

**Figure 4:**
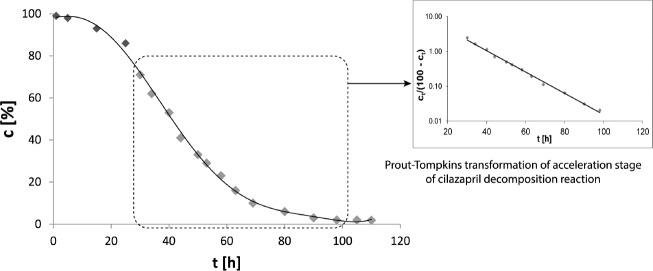
The degradation kinetics of pure CIL – autocatalytic Prout-Tompkins reaction

The kinetic parameters of the stability of pure CIL are derived from our previous studies with CIL in solid state (24-26). According to the provided literature data CIL undergoes a rapid degradation described by the autocatalytic reaction model (Figure 4). In the current study it has been proven that the presence of PVP changes the reaction kinetics to the first order. This alternation can be considered as a benefit since the process of CIL decay in the presence of PVP becomes more predictable, increasing thereby safety of CIL clinical use. The reaction is one-phase instead of three-phase (slow initiation, rapid acceleration and slow termination) as demonstrated for pure CIL and there is no danger of its rapid acceleration in the presence of degradation product or secondary to improper storage in home medicine cabinet (24). Furthermore, the corresponding degradation rate constants k for pure CIL and CIL in CIL/PVP formulations differs significantly. Under the experimental conditions of increased RH = 75% and T = 70 ° C, k for pure CIL equaled (1.217 ± 0.059) 10^-7 ^s^-1 ^(t_0,5_ = 9,5 days) while for CIL/PVP formulation it was two orders of magnitude lower,* i.e.*, (7.863 ± 0.045) 10^-9 ^s^-1 ^(t_0,5_ = 1020 days) for physical mixture and (1.000 ± 0.022) 10^-9 ^s^-1 ^(t_0,5 = _1020 days) for solid dispersion (*p* < 0.05). This indicates that the addition of PVP significantly improved the stability of CIL. The half-life of CIL in the formulation with PVP increased over 33 months.

The effect of temperature on CIL/PVP degradation rate was studied by conducting the reaction at five different temperatures under RH ∼76.4%. For each series of CIL/PVP solid dispersions and CIL/PVP physical mixtures, a degradation rate constant (k) was elucidated and the natural logarithm of each k was plotted against the reciprocal of the corresponding temperature to fulfil the Arrhenius relationship. Then, the energies of activation (E_a_) of the studied reactions were established using the following formula: 

ln k_i_ = lnA – E_a_/RT

where k_i_ - reaction rate constant (s^-1^), A - frequency coefficient, E_a_ - activation energy [J mol^-1^], R - universal gas constant (8.3144 J K^-1^ mol^-1^), T - temperature (K). Furthermore based on the transition state H^¹ ^theory, enthalpy of activation (DH^¹^) and entropy of activation (ΔS^¹^) under temperature 20 °C and RH ˜76.4% were determined using the following equations:

Ea = -a · R

Ea = ΔH^¹^ + RT

ΔS^¹^ = R lnA – ln KT/h

where: a is the slope of ln ki = f(1/T) straight-line, A is a frequency coefficient, Ea is activation energy (J mol-1), R is universal gas constant (8.3144 J K-1 mol-1), T is temperature (K), ΔS^¹^ is entropy of activation (J K-1 mol-1), ΔH^¹^ is enthalpy of activation (J mol-1), K is Boltzmann constant (1.3806488(13) × 10−23J K−1), h is Planck’s constant (6,62606957(29) 10–34 J s) 16. The calculated Ea describes strength of the cleaved bonds in CIL molecule during degradation. Its decreasing values with temperature together with the increasing k values clearly indicate that heating compromises the stability of CIL in the studied formulations with PVP. Interestingly, the obtained result Ea = 166.49 ± 20.8 kJ/mol for pure CIL is high when compared to other structurally-related ACE-Is: imidapril 104.35 kJ/mol, moexipril 116.96 kJ/mol, benazepril 121.16 kJ/mol, perindopril 124.22 kJ/mol, quinapril 133.62 kJ/mol and enalapril 149.11 kJ/mol (30, 32, 34-36 and 39). However, the results for other ACE-Is in their solid dispersions with PVP are not available. As evidenced in Table 3 the presence of PVP increased the stability of CIL and its resistance to heating. Pure CIL required the activation energy equaling Ea = 166.49 ± 20.8 kJ/mol to start do decompose, however in the presence of PVP the essential energetic input increased to 202.36 ± 40.8 kJ/mol for CIL/PVP physical mixture and 276.33 ± 48.9 kJ/mol for CIL/PVP solid dispesrion 17 (*p *< 0,05), [t(α;df) < | t | H1: a1 ≠a2]. These observations suggest that in the presence of PVP the strength of chemical bonds in CIL increases significantly and therefore more energy is needed for their clevage. Furthermore, we found that the CIL/PVP solid dispersion was more stable than the CIL/PVP physical mixture as its Ea of degradation was higher. The difference was statistically relevant (*p *< 0.05) suggesting that the evaporation method of preparing CIL/PVP solid dispersions is more favorable. The positive ΔH≠ in each experiment indicates the endothermic character of the observed reactions, which means that there is a need for constant energy supply during the formation of the activated complex from the participating reagents. Furthermore, the ΔS≠ describes the thermodynamic equilibrium of the system while forming activated complex and provides clues about the molecularity of the rate-limiting step of the reaction. The obtained positive results (Table 3) indicate that the activated complex is less constrained than the individual reagents. Furthermore, as a rule: the higher Ea the higher ΔS≠, while larger ΔS≠ implies more favorable configurational driving force for the reaction. The effect of humidity on the stability of CIL in the formulation with PVP was also investigated at 90 °C, within RH range of 25.0%-76.4%. The natural logarithm of the measured degradation rate constants was plotted *versus *the corresponding RH value and the following linear relationships were obtained:

Pure CIL: ln ki = ax + b = (0.036 ± 0.006) RH% - (-13.526 ± 0.23)

CIL/PVP solid dispersion ln ki = ax + b = (0.024 ± 0.009) RH% - (-14.510 ± 0.57)

CIL/PVP physical mixture ln ki = ax + b = (0.023 ± 0.002) RH% - (-14.520 ± 0.15).

**Figure 5:**
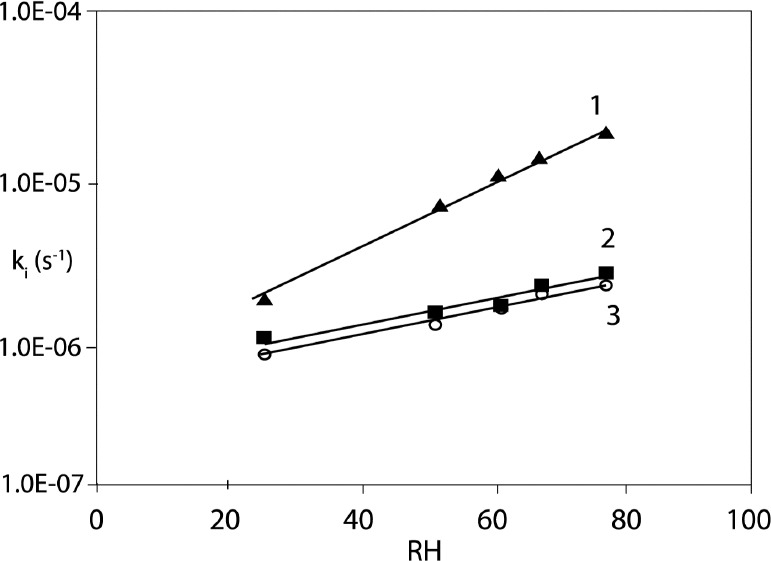
The effect of RH variations on CIL stability (1) pure CIL (2) CIL/PVP solid dispersion, (3) CIL/PVP physical mixture. RH range 25.0%–76.4% (T = 90 °C)

The detailed data on the influence of RH on CIL/PVP formulation stability are demonstrated in Table 4. Our results clearly show that the increase of RH accelerates the degradation of CIL and the appropriate degradation rate constants increase exponentially with RH (Figure 5). The sensitivity to RH also differs significantly between pure CIL and CIL/PVP formulations. In our study this parameter is described by the slope of the lnk = aRH(%) + b plot*. *For pure CIL it was estimated as a = 0.036 ± 0.006, which was significantly higher than the corresponding values for CIL/PVP formulations, *i.e.*, (a = 0.023 ± 0.002) in physical mixture and (a = 0.024 ± 0.009) in solid dispersion (*p *< 0,05), [t(α;df) < | t | H1: a1 ≠a2]. These numbers suggest that PVP protects CIL from moisture and delays its degradation. When compared to other structurally-related ACE-Is CIL occurs as highly sensitive to moisture (a = 0.036). For example, for benazepril a = 0.02, for enalapril a = 0.02 and for imidapril a = 0.03 therefore these drugs are more stable under increased RH (30, 32 and 36). Only moexipril (a = 180.07) is more fragile than CIL; however, its successful stabilization with glyceryl behenate was previously reported (35, 38).

The joint effect of both temperature and RH on the stability of CIL in PVP/CIL mixtures can be depicted by the modified Arrhenius equation which takes the following form:

CIL/PVP solid dispersion: lnki = (-48950 ± 1546) (1/T) + (0,013 ± 0,004) (RH%) + (120,9 ± 4,2)

CIL/PVP physical mixture: lnki = (-36062 ± 1685) (1/T) + (0,012 ± 0,004) (RH%) + (85,4 ± 4,9)

**Figure 6:**
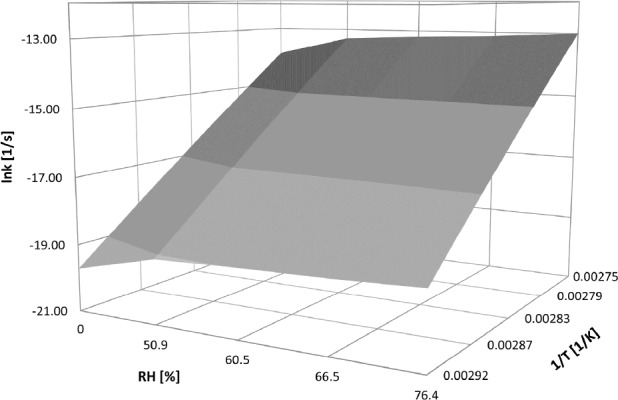
The degradation surface for CIL/PVP physical mixture

Then, based on the established linear semilogarithmic relationships f(RH) = lnki and f(1/T) = lnki the surface of the studied CIL/PVP formulations degradation was constructed (Figure 6). 

It demonstrates the three-dimensional relationship between logarithm of degradation rate constants versus relative humidity and the reciprocal of temperature. The provided equations enable prediction of the degradation rate constant for CIL/PVP mixtures using easy-to-measure values of drug storage conditions.

**Figure 7:**
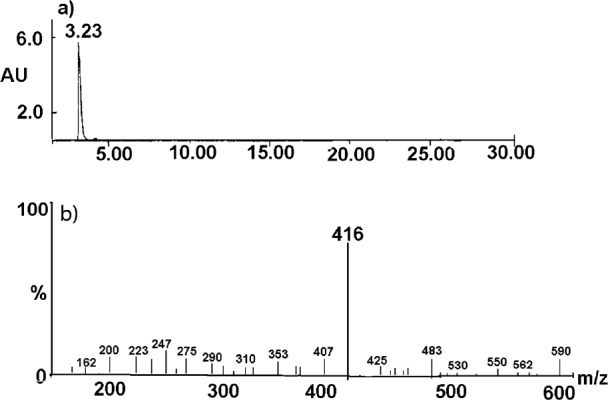
HPLC-MS spectrum for the undegraded CIL/PVP solid dispersion sample. The results for CIL/PVP physical mixture were analogous. The description in the text

**Figure 8:**
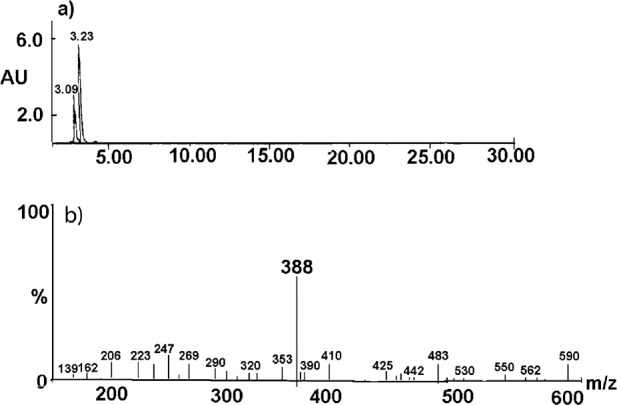
HPLC-MS spectrum for the CIL/PVP solid dispersion after the exposure to stress conditions. The results for CIL/PVP physical mixture were analogous. The description in the text

The degradation product of the observed CIL decay was identified as cilazaprilat based on the analysis on the obtained HPLC-MS spectra (Figures 7 and 8). 

**Figure 9:**
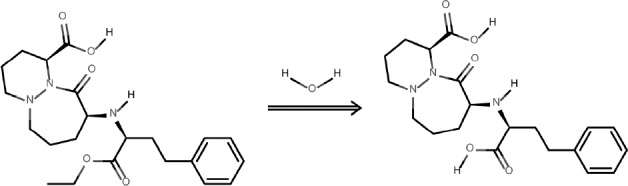
The mechanism of CIL degradation in the presence of PVP

The undegraded sample of CIL/PVP mixtures (kinetic test t = 0) provided a chromatogram with only one peak at tR = 3.23 min (Figure 7A). The corresponding MS spectrum (Figure 7B) demonstrates the signal described as pseudomolecular ion [M - H+]- at m/z equaling 416, indicating a compound with molecular mass 417 u that corresponds to cilazapril anhydrous. The analysis of the degraded sample of CIL/PVP mixtures (RH = 76, 4% T = 353 K) provided a chromatogram with two peaks at tR = 3.23 min (CIL) and tR = 3.09 min (the degradation product) (Figure 8A). The corresponding MS spectrum for the degradation product (Figure 8B) demonstrates the signal described as pseudomolecular ion [M - H+]- at m/z equaling 388, indicating a compound with molecular mass 389 u that can be attributed to cilazaprilat. Therefore, we suggest that under the conditions of our kinetic study (RH 76, 4%, T = 353 K) CIL undergoes a single reaction of ester bond hydrolysis producing cilazaprilat, as shown in Figure 9. This is a typical way of dicarboxylate ACE-Is degradation, which was also demonstrated in our previous studies (24, 21, 26, 31, 32, 35, 36, and 38). The presence of PVP did not influence the degradation pathways of CIL since in its pure form it also undergoes a single reaction of hydrolysis as described elsewhere (24).

**Figure 10:**
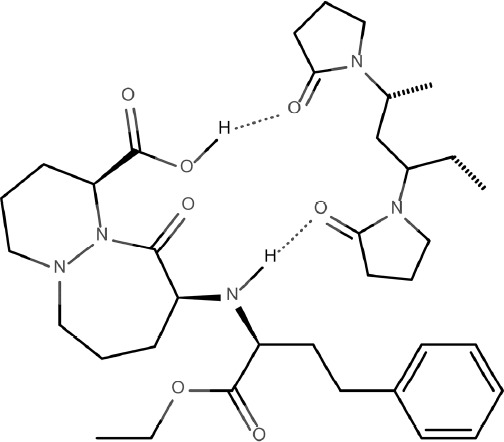
The hydrogen bonding interactions between CIL and PVP

The demonstrated advantageous effect of PVP on the stability of CIL can be explained by the water-scavenging properties of PVP. It is highly probable that this hygroscopic carrier absorbs water molecules from the environment surrounding CIL making them unavailable for the interaction with the drug. This seems additionally relevant given the fact that the only way of CIL degradation is hydrolysis of ester bond. What is more, the interaction by hydrogen bonds between CIL and PVP can be assumed. These bonds occur between the hydrogen bond donor groups of CIL, *i.e.*, secondary amine and hydroxyl (-NH- and –OH) and hydrogen bond acceptor group of PVP - carbonyl (-C=O) as demonstrated in Figure 10. 

The presence of hydrogen bonds can be explained by FT-IR spectra (Figure 1) in which the change of peak shape at 1900 cm^-1 ^(corresponding to carbonyl –C=O bond in PVP) as well as at 3700–3000 cm^-1^ (corresponding to hydroxyl –OH and secondary amine –NH- in CIL) in the spectrum for PVP/CIL was reported.

**Table 1:** Linearity parameters of the RP-HPLC stability indicating method

**Parameters**	**CIL/PVP physical mixture**	**CIL/PVP solid dispersion**
Linearity range mg mL-1	0.08 – 0.4	0.08 – 0.4
Regression equations	Y = a c +b	
Slope a ±	4.955 ± 0.239	4.941 ± 0.225
Sa	1.039	1.028
*Intercept b ±	0.0237 ± 0.0059	0.0221 ± 0.0067
Sb	0.0258	0.0239
Regression coefficient	0.998	0.998
Sy	0.0377	0.0371
LOD mg mL-1	0.025	0.025
LOQ mg mL-1	0.073	0.073

* Intercept b from equation y = ac + b was statistically insignificant (*t*-Student test, α = 0.05); S , S , S standard deviation of slope a, intercept b and y, respectively.

**Table 2:** Precision and recovery data for the RP-HPLC stability indicating method

**Drug**	**Concentration (mg mL** **-1** **)**	**Amount found**	**Recovery (%)**	**Mean Recovery ± SD (%)**
**Intra-day precision**				
	0.08	0.0803	100.75 ± 0.58	100.40 ± 0.89
	0.2	0.2010	100.50 ± 1.09	
	0.4	0.3998	99.95 ± 0.99	
**CIL/PVP physical mixture**		Inter-day precision		
	0.08	0.0801	100.25 ± 0.66	100.08 ± 0.73
	0.2	0.1996	99.80 ± 0.87	
	0.4	0.4008	100.20 ± 0.65	
**Intra-day precision**				
	0.08	0.0801	100.25 ± 0.69	100.28 ± 0.52
	0.2	0.2009	100.45 ± 0.45	
	0.4	0.4006	100.15 ± 0.41	
**CIL/PVP solid dispersion**		Inter-day precision		
	0.08	0.0805	101.25 ± 0.78	100.33 ± 0.65
	0.2	0.1998	99.90 ± 0.65	
	0.4	0. 3993	99.83 ± 0.53	

**Table 3:** The kinetic and thermodynamic stability data for pure CIL and CIL/PVP formulations

**Temp. (°C)**	**k ± ∆k [s** **-1** **]**	**r**	**Linear Arrhenius relationship f (1/T) = lnk**	**Thermodynamic parameters**
**Pure CIL (6)**				
70	(1.217 ± 0.059) 10-7	-0.998		
75	(7.607 ± 0.418) 10-7	-0.998	a = -20025.29 ± 2500.3 sa = 785.75 b = 44.21 ± 7.2 sb = 2.26 r = 0.998	Ea [kJ/mol] 166.49 ± 20.8 H [kJ/mol] 164.02 ± 23.3 S [J/mol∙K] 122.68 ± 185.1
80	(1.662 ± 0.129) 10-6	-0.994		
85	(2.963 ± 0.202) 10-6	-0.994		
90	(1.941 ± 0.106) 10-5	-0.997		
**CIL/PVP physical mixture**				
70	(7.863 ± 0.045) 10-9	-0.997		
75	(3.852 ± 0.319) 10-8	-0.999	a = -24339.09 ± 4892.4 sa = 1762.39 b = 54.12 ± 14.1 sb = 5.07 r = 0.992	Ea [kJ/mol] 202.36 ± 40.8 H [kJ/mol] 199.94 ± 49.1 S [J/mol∙K] 205.14 ± 127.8
80	(9.672 ± 0.426) 10-8	-0.996		
85	(4.008 ± 0.198) 10-7	-0.995		
90	(3.009 ± 0.226) 10-6	-0.998		
**CIL/PVP solid dispersion**				
70	(1.000 ± 0.022) 10-9	-0.996		
75	(9.886 ± 0.114) 10-9	-0.998	A = -33236.08 ± 5881.8 sa = 5881.47 b = 78.81 ± 19.5 sb = 6.09 r = 0.993	Ea [kJ/mol] 276.33 ±48.9 H [kJ/mol] 273.86 ± 51.3 S [J/mol∙K] 410.34 ± 104.2
80	(4.036 ± 0.246) 10-8	-0.995		
85	(3.220 ± 0.099) 10-7	-0.998		
90	(3.012 ± 0.316) 10-6	0.997		

**Table 4:** The effect of humidity on the stability of CIL in pure and CIL co-formulated with PVP at 90 °C

**T = 363 K – influence of air relative humidity on CIL decomposition**			
	**Pure CIL (6)**	**CIL/PVP solid dispersion**	**CIL/PVP physical mixture**
**RH%**		**k ± ∆k [1/s]**	
25.0 %	(3.270 ± 0.241) 10-6	(9.460 ± 0.822) 10-7	(9.010 ± 0.664) 10-7
50.9 %	(7.922 ± 0.964) 10-6	(1.522 ± 0.514) 10-6	(1.555 ± 0.419) 10-6
60.5 %	(1.189 ± 0.068) 10-5	(1.999 ± 0.168) 10-6	(2.079 ± 0.281) 10-6
66.5 %	(1.583 ± 0.143) 10-5	(2.883 ± 0.467) 10-6	(2.32 3 ± 0.415) 10-6
76.4 %	(1.941 ± 0.106) 10-5	(3.041 ± 0.143) 10-6	(3.010 ± 0.331) 10-6
**linear relationship lnk = f(RH)**			
a	0.036 ± 0.006	0.024 ± 0.009	0.023 ± 0.002
sa	0.002	0.002	0.0008
b	-13.526 ± 0.23	-14.510 ± 0.57	-14.520 ± 0.15
sb	0.102	0.181	0.481
r	0.997	0.976	0.998

The parameters: a, Sa, b, Sb and r were calculated by least square regression and are standing for slope, standard deviation of the slope, intercept, standard deviation of intercept and correlation coefficient, respectively.

The hydrogen bonding interactions in PVP/CIL mixtures could additionally increase physical stability of the drug by reducing molecular mobility of the system (17). These observations clearly indicate that PVP can be considered as a stabilizer for CIL in its solid dosage forms.

## Conclusion

The physical mixtures of CIL and a biopolymer – PVP can be achieved successfully by milling and evaporation technique while FT-IR is applicable for the identity confirmation of such forms. 

The stability of CIL in CIL/PVP solid dispersion and CIL/PVP physical mixture can be evaluated by the use of RP-HPLC method, thanks to its linearity, precision, selectivity, and sensitivity. The degradation of CIL in the CIL/PVP dispersions under the conditions of increased temperature and relative humidity RH = 76, 4% proceeds according to first-order kinetics.

 The degradation rate constants for CIL in CIL/PVP forms are significantly lower than that for pure CIL. The Ea for pure CIL is lower than the corresponding value for CIL in CIL/PVP formulations. The sensitivity of CIL to moisture in the presence of PVP is decreased when compared to pure CIL. 

Furthermore, it was shown that in the presence of PVP the conversion of cilazapril into cilazaprilat was halted. All the above data indicate that the solid dispersions with PVP made CIL stable and can therefore PVP can be considered as an excipient for its pharmaceutical formulations.
